# Do smoking, nutrition, alcohol use, and physical activity vary between regions in Germany? - results of a cross-sectional study

**DOI:** 10.1186/s12889-020-8352-2

**Published:** 2020-03-13

**Authors:** Josefine Atzendorf, Christian Apfelbacher, Elena Gomes de Matos, Kirsten Lochbühler, Daniela Piontek, Nicki-Nils Seitz, Ludwig Kraus

**Affiliations:** 1 Max Planck Institute for Social Law and Social Policy, Amalienstraße 33, 80804 Munich, Germany; 2 IFT Institut für Therapieforschung, Munich, Germany; 3 Department of Medical Sociology, Institute of Epidemiology and Preventive Medicine, University of Regensburg, Regensburg, Germany; 4 Institute of Social Medicine and Health Systems Research, Otto von Guericke University Magdeburg, Magdeburg, Germany; 5 Department of Public Health Sciences, Centre for Social Research on Alcohol and Drugs, Stockholm University, Stockholm, Sweden; 6 Institute of Psychology, ELTE Eötvös Loránd University, Budapest, Hungary

**Keywords:** Epidemiology, Lifestyle risk factors, Population survey, Regional differences, East Germany, West Germany, South-north gradient, Health risk

## Abstract

**Background:**

Previous studies on lifestyle risk factors mainly focused on age- or gender-specific differences. However, lifestyle risk factors also vary across regions. Aim of the present study was to examine the extent to which prevalence rates of SNAP (smoking, nutrition, alcohol consumption, physical activity) vary between East and West Germany or North and South Germany.

**Methods:**

Data came from the population-representative 2015 Epidemiological Survey of Substance Abuse (ESA) comprising 9204 subjects aged 18 to 64 years. To assess an east-west or south-north gradient, two binary logistic regression models were carried out for each SNAP factor.

**Results:**

The logistic regression models revealed statistically significant differences with higher rates of at-risk alcohol consumption and lower rates of unhealthy nutrition in East Germany compared to West Germany. Significant differences between North and South Germany were found for at-risk alcohol consumption with higher rates of at-risk alcohol consumption in South Germany. Daily smoking and low physical activity were equally distributed across regions.

**Conclusions:**

The implementation of measures reducing at-risk alcohol consumption in Germany should take the identified east-west and south-north gradient into account. Since the prevalence of unhealthy nutrition was generally high, prevention and intervention measures should focus on Germany as a whole instead of specific regions.

## Background

Smoking, unhealthy nutrition, at-risk alcohol consumption and physical inactivity (SNAP) are known as lifestyle risk factors which are related to non-communicable diseases such as cardiovascular diseases, cancer, or chronic respiratory diseases [1, 2]. According to the Global Burden of Disease Study non-communicable diseases accounted for 72.3% premature deaths worldwide in 2016 [3]. The economic costs for Germany due to diseases caused by drinking and smoking were estimated at about 26–79 billion euro per year, respectively [4].

The risk of SNAP varies by sociodemographic factors such as age, gender or education, and across geographical or political regions. Some studies reported an east-west [5] or south-north gradient [6] in regard to at-risk alcohol use in the German population, whereas another study only found south-north differences in drinking styles [7]. For instance, in Southern federal states beer was consumed more often than wine or spirits compared to Northern federal states, but there were no regional differences regarding the prevalence of alcohol-related problems [7]. Völzke and colleagues [8] reported that young people in East Germany were more likely to smoke than in West Germany and the onset of smoking in middle-aged women was later in East Germany compared to women of the same age in West Germany. In the adult population, smoking was found to be more prevalent in the Northern compared to the Southern federal states [9]. Furthermore, nutrition was also found to vary across federal states [10]. For example, eating bread was more often reported by women in the Eastern federal states compared to women in the Western federal states, while both genders in East Germany were found to eat more fruits, vegetables, fats, sausages, meat-based products, but less fish and cereal products than in West Germany [10]. Physical activity among children was reported to be lower in East Germany [5]. Differences in terms of physical activity between West and East Germany might be attributed to the lower number of sport facilities in East Germany [5]. The amount of non-organized physical activity in the adult population, e.g. leisure time activities, did not differ between regions [5].

After the separation of Germany in East and West after World War II and its reunion in 1989, the federal states still vary by sociodemographic and macroeconomic characteristics. The Federal Ministry for Economic Affairs and Energy reported differences between East and West in terms of economic power [11]. For instance, in 2016 the gross domestic product per capita was 28,000 € in East Germany in comparison to 39,000 € in West Germany [12]. An explanatory factor for the still existing difference in economic power is the absence of larger companies in East Germany which is accompanied among others by a lower export rate [11]. For instance, not a single East German company is listed in the stock market index DAX-30 [11]. Furthermore, a lower population density in East Germany might also be an influencing factor of the lower economic power in East Germany [11].

The Hamburg Institute of International Economics (HWWI) [13] also reported a south-north gradient in Germany regarding economic indicators. For example, the Southern federal states Bavaria and Baden-Wuerttemberg showed a higher economic growth compared to the Northern federal states, especially after the financial crisis in 2009 [13]. Additionally, the Southern federal states Bavaria and Baden-Wuerttemberg had the lowest unemployment rates in 2015 (2.6 and 3.2%) whereas the federal states Berlin and Saxony-Anhalt in the north of Germany had the highest unemployment rates with 7.9 and 7.5%, respectively [14]. Furthermore, debts of the Northern states (estimated at 371 billion euros) are twice as high as the debts of the Southern states (170 billion euros) [15]. The south-north divide in Germany can be explained by historical differences in mentalities due to traditional structures in the agricultural sector. In North Germany the agricultural economy was dominated by large landowners until the nineteenth century, whereas South Germany was dominated by peasants. Therefore, the economic dependence was more pronounced in North Germany and the developments of self-initiative and entrepreneurial actions were outweighed by a subordinated mentality. On the other side, the higher density of enterprises and more widespread self-employment in South Germany meant that stronger competition could be established at an early stage, rewarding entrepreneurial thinking and self-reliance. This tradition of self-reliance in combination with the peasant ethos is seen as the basis for the later rise of the South [13, 16]. Furthermore, the transition to the knowledge society can be seen as the initial spark, since autonomous actions gained importance [13, 16].

In previous studies on lifestyle risk factors at population level, the focus was generally on age- or gender-specific differences. However, as described above, there are indicators that lifestyle risk factors also vary across regions. The aim of the present study is therefore to examine if the regional differences of SNAP are still valid. Additionally, since previous literature postulated the assumption that the south-north gradient of economic differences could aggravate in the future and replace the east-west gradient [13], the present study will also investigate the extent to which there are increased differences in the distribution of SNAP indicators between North and South Germany compared to East and West Germany. The findings will provide the possibility to develop or adjust health policy strategies for lifestyle risk factors by considering regional variabilities.

## Methods

### Study design and sample

The 2015 Epidemiological Survey of Substance Abuse (ESA 2015) served as database. Based on a cross-sectional design the ESA has been assessing substance use among the general population in Germany at regular intervals since 1980. In 2015, a random two-stage procedure was used for sampling: 254 sample points (cities or communities) followed by a random selection of the target population from population registers. Data were collected by standardized self-administered questionnaires (paper-pencil), telephone interviews, or online questionnaires from 18 to 64 years old individuals living in Germany. The initial sample was divided into a telephone arm and a written arm depending on whether a telephone number could be determined for the respective address. All selected individuals received written correspondence comprising study information, a data privacy statement, an online access code, and an accompanying letter from the German Federal Ministry of Health. Informed consent was given either verbally (telephone arm), by ticking the informed consent box (online questionnaire) or by accepting the conditions (study content, data protection, data processing and storage) in sending back the filled in questionnaire. The procedure had been approved by the ethics committee of the German Psychological Society (DGPs; Reg.-No: GBLK06102008DGPS). The net-response rate was 52.2%. The final sample comprised 9204 study participants, of whom 5090 were female (49.6%) and 4114 were male (50.4%). The average age of the participants was 42.3 years (95%-CI = [42.0; 42.7). For further information on methods and design of the ESA 2015 please see Piontek and colleagues [17].

### Outcome measures

SNAP` were used as outcome measures and all variables were dichotomized (0 = favorable lifestyle behavior; 1 = risky lifestyle behavior). Daily smoking was defined by having smoked at least one cigarette, cigar, pipe or cigarillo per day in the last 30 days. To assess unhealthy diet, a validated food frequency questionnaire of six items was used (LML-6) [18]. Participants were asked how often (1 rarely or never - 5 several times a day) they had eaten low fat milk products, crudités, fresh salads, fresh herbs, fresh fruits or cereal products [18]. Responses to the LML-6 were categorized into healthy diet (index score > = 10) and unhealthy diet (index score < 10) [18]. A quantity-frequency-index was used to assess alcohol consumption. At-risk alcohol consumption in the last 30 days was characterized by an average daily ethanol intake of 12 g or more for women or 24 g or more for men [19, 20]. Physical activity was assessed by asking the participants on how many days per week in the last three months they had been breathless and sweating due to physical activity. If they had been active for at least one day per week, they were asked about the average duration of their physical activity (1 “Less than 10 minutes”, 2 “10 to less than 30 minutes”, 3 “30 to 60 minutes”, 4 “More than 60 minutes”). Based on the recommendations of the American College of Sports Medicine and the American Heart Association, being physical active for less than 30 min per day on five days a week was defined as low physical activity [21].

### Predictors

Two dichotomous variables were generated to divide the 16 German federal states into North (Berlin, Brandenburg, Mecklenburg-Western Pomerania, Saxony-Anhalt, Bremen, Hamburg, Lower Saxony, North Rhine-Westphalia and Schleswig-Holstein), South (Baden-Wuerttemberg, Bavaria, Hesse, Rhineland-Palatinate, Saarland, Saxony and Thuringia), East (Brandenburg, Mecklenburg-Western Pomerania, Saxony-Anhalt, Saxony and Thuringia) and West (Baden-Wuerttemberg, Bavaria, Bremen, Hamburg, Hesse, Lower Saxony, North Rhine-Westphalia, Schleswig-Holstein, Rhineland-Palatinate, Saarland) Germany. The East-West division followed the borders of the former German Democratic Republic (GRD) and the Federal Republic of Germany (FRG) between 1945 and 1989. The classification in North and South Germany was based on population size (same population size in North and South Germany), urbanity (five urban regions each in North and South Germany) and the number of inhabitants from the new Eastern federal states [15].

As sociodemographic information gender, age and education were assessed. Education was assessed based at the International Standard Classification of Education (ISCED) [22] and categorized in three groups: low education, intermediate education, high education.

### Statistical analyses

Two binary logistic regression models were carried out for each SNAP-indicator. Since the same federal states are divided once into East-West and once into South-North, the regions (East-West, South-North) were included in separate regression models to prevent multicollinearity. All predictors were entered at once. The mode of data collection (paper pencil, telephone, online) served as control variable. Cases with missing data were deleted listwise. Weighted prevalence rates and 95%-confidence intervals were calculated. Data was weighted for region, age, gender and education. All analyses were performed in STATA 14 (Stata Corp LP, College Station, TX).

## Results

### Descriptive results

Table 1 and Table 2 depict the prevalence rates for the four SNAP-indicators by region (95%-CI not shown). Daily smoking was equally distributed across regions. Unhealthy nutrition was most prevalent in West Germany (70.6, 95%-CI = [69.4; 71.8]) and least prevalent in East Germany (66.7, 95%-CI = [63.4; 69.8]), but the differences were not significant. East Germany showed the highest prevalence for at-risk alcohol consumption (18.3%, 95%-CI = [16.2; 20.6]), which differed statistically significant from the prevalence in West (14.6, 95%-CI = [13.6; 15.8]) and North Germany (13.9, 95%-CI = [12.7; 15.1]). The prevalence of low physical activity did not differ between the regions.

**Table 1:** Overview of prevalence rates of the four SNAP factors for East (*n* = 1430) and West Germany (*n* = 7429)

SNAP factors	West	East
Daily Smoking	18.5	19.1
Unhealthy Nutrition	70.6	66.7
At-risk alcohol consumption	14.6	18.3
Low physical activity	82.5	82.5

Note. % = Figures represent weighted prevalence rates.

**Table 2:** Overview of prevalence rates of the four SNAP factors for North (*n* = 4447) and South Germany (*n* = 4757)

SNAPfactors	North	South
Daily Smoking	19.6	17.4
Unhealthy Nutrition	70.4	69.4
At-risk alcohol consumption	13.9	16.7
Low physical activity	82.9	82.3

Note.  Figures represent weighted prevalence rates.

### Daily smoking

Women (OR = 0.8, 95%-CI = [0.7; 0.9]) and individuals with a higher education (OR = 0.4, 95%-CI = [0.3; 0.5]) were less likely to smoke at a daily basis. Under the control of sociodemographic factors, there was no statistically significant east-west (Table 3) or south-north gradient (Table 4).

### Unhealthy nutrition

Women (OR = 0.3, 95%-CI = [0.3; 0.4]) and individuals with higher education (OR = 0.7, 95%-CI = [0.6; 0.9]) were less likely to report unhealthy nutrition compared to men and individuals with a lower education. When controlling for sociodemographic variables, a statistically significant east-west gradient was found (Table 3). Individuals in East Germany were less likely to report unhealthy nutrition than individuals in West Germany (OR = 0.8, 95%-CI = [0.7; 1.0]). There was no statistically significant south-north gradient (Table 4).

### At-risk alcohol consumption

Women (OR = 0.8, 95%-CI = [0.6-0.7; 0.90]) were less likely to drink alcohol at a risky level than men. Individuals with higher (OR = 1.5, 95%-CI = [1.1; 1.9]) or intermediate education (OR = 1.5, 95%-CI = [1.1; 2.0]) were more likely to report at-risk alcohol consumption than individuals with lower education. Statistically significant east-west (Table 3) and south-north gradients (Table 4) were found under the control of sociodemographic factors. Individuals in East Germany were more likely to report at-risk alcohol consumption compared to individuals in West Germany (OR = 1.3, 95%-CI = [1.1; 1.5]), and individuals in North Germany were less likely to drink alcohol at a risky level compared to individuals in South Germany (OR = 0.8, 95%-CI = [0.7; 0.9]).

### Low physical activity

Women (OR = 1.5, 95%-CI = [1.3; 1.8]), older people (OR = 1.0, 95%-CI = [1.0; 1.0]) and individuals with higher education (OR = 1.8, 95%-CI = [1.4; 2.4]) were more likely to report low physical activity compared to men, younger people and individuals with lower education. No statistically significant east-west (Table 3) or south-north gradients (Table 4) were found when controlling for sociodemographic variables.

**Table 3:** Estimations of differences for daily smoking, unhealthy nutrition, at-risk alcohol consumption, and low physical activity between East and West Germany

Variables	Daily Smoking		Unhealthy Nutrition		At-risk alcohol consumption		Low physical activity	
	OR	95%-CI	OR	95%-CI	OR	95%-CI	OR	95%-CI
Gender								
Male *(reference)*	–	–	–	–	–	–	–	–
Female	0.80*	[0.68; 0.94]	0.32*	[0.29; 0.36]	0.76*	[0.64; 0.89]	1.53*	[1.28; 1.83]
Age	1.00	[1.00; 1.01]	1.00	[0.99; 1.00]	1.00	[1.00; 1.01]	1.01*	[1.00; 1.01]
Education								
Low *(reference)*	–	–	–	–	–	–	–	–
Intermediate	0.84	[0.64; 1.10]	1.11	[0.90; 1.35]	1.52*	[1.14; 2.02]	1.27	[0.99; 1.64]
High	0.39*	[0.30; 0.52]	0.73*	[0.60; 0.90]	1.45*	[1.09; 1.94]	1.85*	[1.43; 2.38]
Region								
West *(reference)*	–	–	–	–	–	–	–	–
East	1.11	[0.91; 1.35]	0.82*	[0.70; 0.95]	1.25*	[1.05; 1.48]	0.94	[0.79; 1.11]

Note. *reference* = reference group; *p < .05; OR = Odds ratio; 95%-CI = 95% confidence intervals

**Table 4:** Estimations of differences for daily smoking, unhealthy nutrition, at-risk alcohol consumption, and low physical activity between North and South Germany

Variables	Daily Smoking		Unhealthy Nutrition		At-risk alcohol consumption		Low physical activity	
	OR	95%-CI	OR	95%-CI	OR	95%-CI	OR	95%-CI
Gender								
Male *(reference)*	–	–	–	–	–	–	–	–
Female	0.80*	[0.68; 0.94]	0.32*	[0.29; 0.36]	0.76*	[0.65; 0.90]	1.53*	[1.28; 1.83]
Age	1.00	[1.00; 1.01]	1.00	[0.99; 1.00]	1.00	[1.00; 1.01]	1.01*	[1.00; 1.01]
Education								
Low *(reference)*	–	–	–	–	–	–	–	–
Intermediate	0.85	[0.66; 1.11]	1.10	[0.90; 1.34]	1.51*	[1.14; 2.01]	1.27	[0.99; 1.64]
High	0.40*	[0.31; 0.52]	0.72*	[0.59; 0.89]	1.46*	[1.09; 1.94]	1.84*	[1.43; 2.38]
Region								
South *(reference)*	–	–	–	–	–	–	–	–
North	1.15	[0.97; 1.36]	1.06	[0.95; 1.18]	0.80*	[0.69; 0.92]	1.05	[0.90; 1.23]

Note. *reference* = reference group; *p < .05; OR = Odds ratio; 95%-CI = 95% confidence intervals

## Discussion

The purpose of this paper was to examine if the four SNAP indicators vary between East and West or between North and South Germany. We found regional differences for at-risk alcohol consumption and unhealthy nutrition, with higher prevalence for at-risk alcohol consumption in East and South Germany and lower prevalence for unhealthy nutrition in East Germany. Daily smoking and low physical activity were equally distributed in the German population.

Men were more likely to smoke daily, show unhealthy nutrition and at-risk alcohol consumption, whereas women were more likely to exhibit low physical activity. These findings are coherent with the existing literature [23, 24]. Younger age was not associated with daily smoking, unhealthy nutrition and at-risk alcohol consumption, but with low physical activity. Previous findings on associations with age and SNAP factors were also inconsistent [23, 24]. Furthermore, in this study higher educated people smoked less often daily and reported unhealthy nutrition less often but were more likely to show at-risk alcohol consumption and low physical activity. According to the research literature, lower education is associated with a number of lifestyle risk factors, but higher education often seems to be associated with more alcohol consumption [23–25]. People with higher education might be better informed about the adverse health effects of smoking compared to people with lower education, but the general acceptance of alcohol as a part of the German culture might lead to a lower awareness of the adverse health effects of drinking [26, 27]. Additionally, people with higher education might have jobs which allow for less time for physical activity [27].

The regional differences regarding the four SNAP factors were independent of the socioeconomic factors. The present study did not find any regional differences regarding daily smoking. In contrast, previous literature reported that younger individuals in East Germany were more likely to smoke than younger individuals in West Germany [8]. A sensitivity analysis (not shown in the results) did not find an east-west gradient for age on daily smoking. In the past years, a number of policy measures regarding tobacco consumption such as an increase of taxes, ban of public advertisements, and the implementation of non-smoking policies have been realised. In consequence, the number of smokers has been overall decreasing in the German population [28]. Despite the declining prevalence smoking continues to be a public health issue in Germany and Germany ranks second last in the implementation of tobacco control policies compared to 36 countries [29]. Prevention and intervention measures for reducing smoking and smoking-related harm should focus on the whole country instead of specific regions.

Further, the present study found an east-west gradient regarding unhealthy nutrition with individuals in East Germany showing a lower risk for unhealthy nutrition. Although, previous literature also reported regional differences in the consumption of certain foods, regional differences for unhealthy or healthy nutrition were not reported [5]. For the assessment of unhealthy nutrition, a food frequency questionnaire (LML-6) consisting of six items was used due to limited space in the survey [18]. Even though the LML-6 was validated [18], six items might be insufficient to assess nutrition comprehensively. Furthermore, it is conceivable that it might not be that crucial which food is eaten, but more importantly which nutrition habit is practiced [5] (for instance, intuitive eating). However, since the prevalence of unhealthy nutrition was generally high (ca. 70%), prevention and intervention measures should focus on the whole country instead of specific regions. Many prevention and intervention measures in Germany solely focus on children (for instance, healthier food choices in school, getting a “nutrition license”) [30], whereas other countries address the whole population by implementing taxes on sugary food (Hungary) or sugary drinks (Great Britain) [31, 32]. Based on the research literature, taxation on certain groceries can promote healthier nutrition [31, 33]. This year, the German government plans to implement a “Nutri-score” on grocery packages [34]. The Nutri-score aims to provide easily accessible information about the nutritional value of a certain food to the customers by using a coloured scale (green = good nutritional value; red = adverse nutritional value) [34]. However, companies will be allowed to implement the Nutri-Score on a voluntarily basis [34].

The present results of regional differences in at-risk alcohol consumption are coherent with previous studies, which also reported a south-north gradient [6] as well as an east-west gradient for men aged 40 to 69 with higher prevalence rates for East and North Germany [5]. As stated above, one further study did not find regional differences in regard to at-risk alcohol consumption [7]. The contrary results of the present paper can be explained by methodological differences between the two studies and different approaches regarding the division of the federal states in North and South Germany. Previous research reported differences regarding alcohol-attributed mortality between East and West Germany, with East Germany showing a higher mortality of alcohol induced diseases compared to West Germany [35, 36]. However, the fatality number of alcohol induced diseases in the South was lower compared to the North of Germany, even though the Southern federal states showed a higher prevalence of at-risk alcohol consumption [35, 36].

Historical differences in alcohol consumption between East and West Germany might explain the east-west gradient for at-risk alcohol consumption. In the 1980s, the German Democratic Republic (GDR) led the world ranking list in per capita consumption of liquor [26]. During the time of the GDR (1945–1989), alcohol had many different functions [26]. It was used as a currency, medium of exchange and self-medication [26]. Furthermore, the social function of alcohol was by far the most important one: in the GDR, collective drinking was seen as a mean to generate feelings of companionship and attachment [37]. Therefore, established drinking patterns had the function to stabilize, harmonize, and deepen social ties [37]. The historical differences between East and West Germany could have led to the establishment of different cultural norms regarding at-risk alcohol consumption. The south-north gradient regarding at-risk alcohol consumption can be explained by different drinking styles between the federal states [7]. For example, federal states in South Germany (Hesse, Saxony, Thuringia, Saarland) consumed beer more frequently per month compared to the national average [7]. Additionally, Rhineland-Palatinate and Baden-Wuerttemberg (federal states in South Germany)consumed wine more frequently per month in comparison with the national average [7]. One glass of beer (250 ml) and one glass of wine (100 ml) are containing 10 g of pure alcohol each, which means if women are drinking two glasses of wine or beer daily, they are already consuming alcohol at a risky level. Furthermore, the southern federal states are known for their folk festivals (e.g. Oktoberfest) and public areas (e.g. beer gardens), where it is ‘normal’ to consume alcohol in large quantities. The implementation of prevention and interventions measures regarding the reduction of alcohol consumption should be enlarged in Germany. So far, many campaigns and prevention initiatives focus on children and adolescents (e. g., www.kenn-dein-limit.de, www.null-alkohol-voll-power.de). The World Health Organization reports policy measures (e. g., increase in taxation for alcohol, ban of public advertising) to be very effective for the reduction of alcohol consumption and alcohol related harm [38]. However, policy measures for reducing alcohol consumption have not been sufficiently implemented in Germany [39]. In addition to alcohol policy measures, screenings and short interventions in primary medical care could be implemented with the aim to reduce alcohol consumption [40]. For instance, the S3-guideline for screening, diagnosis and treatment of alcohol-related disorders in primary health care enables practitioners to detect at-risk alcohol consumption in their patients and offer adequate assistance or treatment at an earlier stage.

Previous literature postulated the assumption that the south-north gradient of economic differences could aggravate in the future and replace the east-west gradient [13]. In 2021, the inter-state fiscal adjustment will be discontinued. Until now, in East Germany the economic development of federal states was promoted in order to reduce an economic east-west gradient. It is unknown, if the discontinuation of the inter-state fiscal adjustment will have effects on an economic south-north or east-west gradient and therefore on regional differences of lifestyle risk factors. Future research should assess associations between regional economic developments and health and lifestyle risk factors.

The present study did not find regional differences regarding physical activity. The prevalence of low physical activity was high in all regions (ca. 82%), which leads to the conclusion that prevention and intervention measures should focus on the whole population instead of specific regions. The ESA survey only assessed moderate-intense physical activity and did not assess vigorous-intense physical activity, which might have led to lower rates of physical activity in the present sample.

### Limitations and strengths

The results of the present study are further limited, because of the collection of self-reported data which might be biased due to socially desirable answers [17]. Additionally, certain subgroups, which are known for increased rates of substance consumption, i.e. homeless people or inmates are not reached by the sampling [41]. Berlin was not included in the analyses between East and West Germany, because a separation in West and East Berlin was not possible. Sensitivity analyses were conducted by assigning Berlin to West Germany, but the regression analyses did not reveal diverging results. Besides the reported limitations, the ESA 2015 provides a big sample of representative data for the general adult population in Germany including data on all four SNAP factors for the first time.

## Conclusions

Even three decades after the reunion of Germany, the present study still reports an east-west gradient regarding alcohol consumption and nutrition. It furthermore revealed a south-north gradient for alcohol use, but not for other lifestyle risk factors. The results of the present study can lead to a better understanding of the social epidemiology of lifestyle risk factors in Germany and thus provide starting points to develop or adjust health policies for reducing lifestyle risk factors by considering regional variabilities. Regional differences for at-risk alcohol consumption should be considered for the implementation of prevention or intervention measures for the reduction of alcohol consumption or alcohol-related harm. For smoking, unhealthy nutrition and low physical activity prevention and intervention measures should focus on the whole population. The present study indicates that the supply situation of health-promoting strategies reducing lifestyle risk factors does not seem to be sufficient. The monitoring of lifestyle risk factors and their regional distribution should be continued as a basis for planning and improving measures regarding a reduction of lifestyle risk factors, and as a result, their economic costs for the society.

## Data Availability

All datasets of the Epidemiological Survey of Substance Abuse (since 1980) are available for scientific purposes at GESIS Leibniz Institute for Social Research and can be requested there. A permission in order to access the data must be obtained in order to access the data by the IFT Institut für Therapieforschung in Munich, Germany.

## Abbreviations

CI: Confidence interval
ESA: Epidemiological Survey of Substance Abuse
GDR: German Democratic Republic
ISCED: International Standard Classification of Education
LML-6: Food frequency questionnaire (original language (German) = Lebensmittelliste)
OR: Odds ratio
SNAP: Smoking, unhealthy nutrition, at-risk alcohol consumption and physical inactivity
